# Clinical Impact of Reporting Coronary Artery Calcium Scores of Non-Gated Chest Computed Tomography on Statin Management

**DOI:** 10.7759/cureus.14856

**Published:** 2021-05-05

**Authors:** Nathan T Douthit, Nicole Wyatt, Brandon Schwartz

**Affiliations:** 1 Internal Medicine, East Alabama Medical Center, Opelika, USA; 2 Internal Medicine, Brookwood Baptist Medical Center, Birmingham, USA; 3 Radiology, Brookwood Baptist Medical Center, Birmingham, USA

**Keywords:** coronary artery calcium score, ascvd risk, statins, non-gated chest ct

## Abstract

Introduction

Coronary artery calcium (CAC) scoring is used as a screening tool for patients with intermediate 10-year arteriosclerotic cardiovascular disease (ASCVD) risk. Results obtained on non-contrast non-gated chest CT (ngCCT) correlate well to those obtained on gated CTs. This paper aims to determine how the routine reporting of CAC scores on ngCCT scans with ASCVD risk of less than 12.5% would change statin management.

Methods

Data of all patients scanned on a single CT scanner during a four-month window were reviewed. A total of 521 eligible scans were identified. After removing duplicate scans and scans from patients who were not in the age range of 40-75 years, 370 scans remained. Patients were excluded if they had documented ASCVD, type 2 diabetes mellitus, or low-density lipoprotein (LDL) > 190 mg/dL, or if they had ASCVD risk of greater than 12.5%. Ultimately, 36 scans were included in the study.

Results

Of the 36 patients who qualified, 10 were low-risk (ASCVD risk<5%), 13 were intermediate-risk (ASCVD risk 5-7.5%), and 13 were high-risk (ASCVD risk 7.5%-12.5%). A CAC score of 300 was used as a cutoff for recommending prescribing statins and 0 was used as a cutoff for recommending de-prescribing statins. In 63% of patients (23/36), CAC scoring altered statin recommendations. This included 11/13 (85%) intermediate-risk patients, 6/13 (46%) high-risk patients, and 6/10 (60%) low-risk patients.

Conclusions

Reporting CAC on ngCCTs obtained for other reasons can significantly impact statin prescribing practices. This may improve cost, patient satisfaction, and patient safety.

## Introduction

Globally, an estimated 15.2 million people died from cardiovascular disease (CVD) in 2012, making it the most frequent cause of death worldwide. The majority of those deaths were secondary to coronary artery disease (CAD) [1,2]. In 2019, the American College of Cardiology (ACC) and the American Heart Association (AHA) revised indications for statin therapy [3]. Previous estimates of similar guidelines showed that 56 million adults in the United States would be eligible for statin therapy, with 87.4% of men aged 60-75 years eligible for therapy [4]. Statin over-prescribing is also a problem, as benefit is seen typically at higher arteriosclerotic cardiovascular disease (ASCVD) risk than is recommended in the guidelines [5].

Coronary artery calcium (CAC) scores have been shown to be more effective predictors of CVD than risk factors and scoring systems [6-9]. CAC scoring has also been added to these risk stratifying scores in order to improve their predictive value [10,11]. The current ACC/AHA guidelines recommend statin therapy if CAC is >100 Agatston units (AU) [3]. There is evidence that healthcare costs and patient safety would improve if this recommendation is followed [12-14].

CAC scores are traditionally obtained by ECG-gated non-contrast cardiac CTs (CCTs). However, CAC scores can also be measured on non-gated non-contrast chest CTs (ngCCTs) [15]. There are vastly higher numbers of ngCCTs than CCTs ordered annually: 13.7 million versus 0.7 million in the USA in 2007 [16]. There are multiple studies showing that CAC measured on ngCCT correlates well to CCT, with the same proven prediction for morbidity and mortality. These can be objectively measured by AU or given ordinal measurements by trained radiologists [14,17-20]. Current recommendations include high-intensity statin for CAC > 300 AU and consideration of moderate- to high-intensity statin for CAC > 100 AU [3].

It remains to be seen in clinical practice how often the reporting of CAC scores from ngCCTs would change the management of patients in regard to statin prescribing practices. Many or most ngCCTs are obtained of patients who have type 2 diabetes mellitus (DMII), clinically significant CVD, or low-density lipoprotein (LDL) > 190 mg/dL, all indications for statin use, which would indicate that CAC score reporting may not frequently change recommendations. However, the accuracy of the pooled cohort equation and Framingham risk scores and their ability to be applied to all patient populations has also been questioned [21]. This would indicate that some high-risk patients, as identified using the Framingham score, are overtreated and some low-risk patients may be undertreated. This study seeks to determine the clinical importance of CAC scores when read from ngCCTs measured and whether or not they would change statin recommendations based on 10-year ASCVD risk derived from the pooled cohort equation. All ngCCTs ordered on a single CT scanner were reviewed in order to determine which individuals who are statin ineligible (ASCVD risk < 5%), statin intermediate (ASCVD risk 5%-7.5%), and mildly statin eligible (ASCVD risk 7.5%-12.5%) will be reclassified to a different statin group based on the reporting of their CAC score.

## Materials and methods

Patient selection process

All ngCCTs obtained on a single machine during a four-month period were considered. This included low-dose screening chest CTs, CTs of the chest, abdomen, and pelvis (both with and without contrast), and CTs of the chest (both with and without contrast). Only patients aged between 40 and 75 years at the time of the scan were considered. Patients were excluded if they had a documented history of CVD, DMII, or LDL > 190 mg/dL, as these factors are indications for statin therapy. They were also excluded if there was no documented lipid profile. The remaining patients were then stratified into three groups by the pooled cohort equation: “low risk” (ASCVD risk < 5%), “intermediate risk” (ASCVD risk 5%-7.5%), and “high risk” (ASCVD risk 7.5%-12.5%). This research was reviewed by MetroWest Medical Center IRB and given exempt status.

Image acquisition and scoring

Multiple contiguous axial CT images of the chest were obtained without the administration of intravenous iodinated contrast. These examinations were performed for various indications, oftentimes unrelated to CAD. Therefore, images were acquired without electrocardiogram (EKG) gating. CAC scoring was performed using the TeraRecon Aquarius iNtuition Edition, version 4.4.13.P2 (TeraRecon, Inc., Durham, NC). Regions of interest were drawn around coronary artery calcifications, and a total calcium score was then generated.

Scoring system

Patients were considered statin eligible if AU was >300. They were considered statin ineligible if AU was 0. If the CAC indications conflicted with the ASCVD indications (statin indicated if ASCVD risk is >7.5% and not indicated if <5%), then they were considered reclassified. All patients in the intermediate-risk group were considered reclassified if CAC was >300 or if it was 0. Proportion confidence intervals were calculated to determine the proportion of the population who may have their statin recommendations changed by CAC scoring. Proportion confidence intervals were determined using Microsoft Excel.

## Results

**Figure 1:**
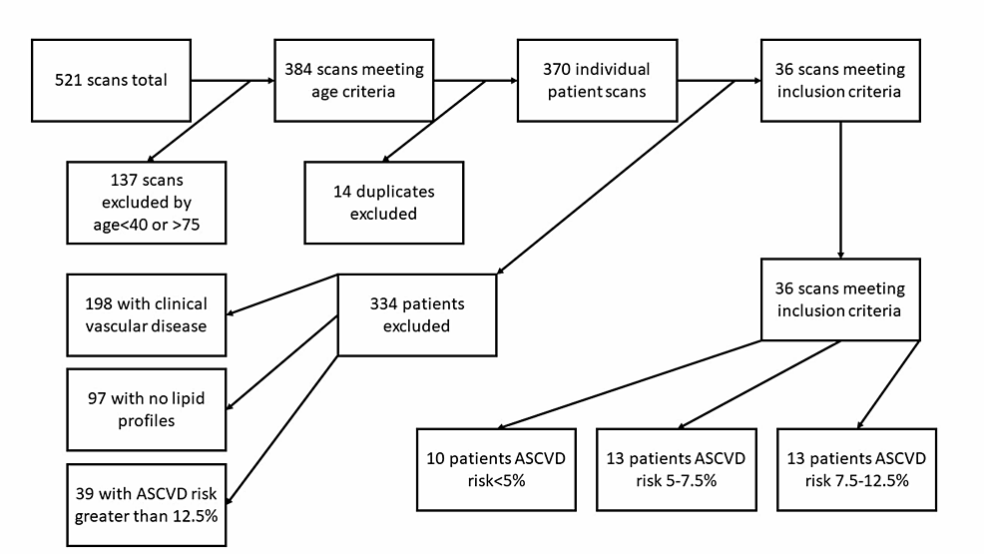
Selection process ASCVD, arteriosclerotic cardiovascular disease

**Figure 2:**
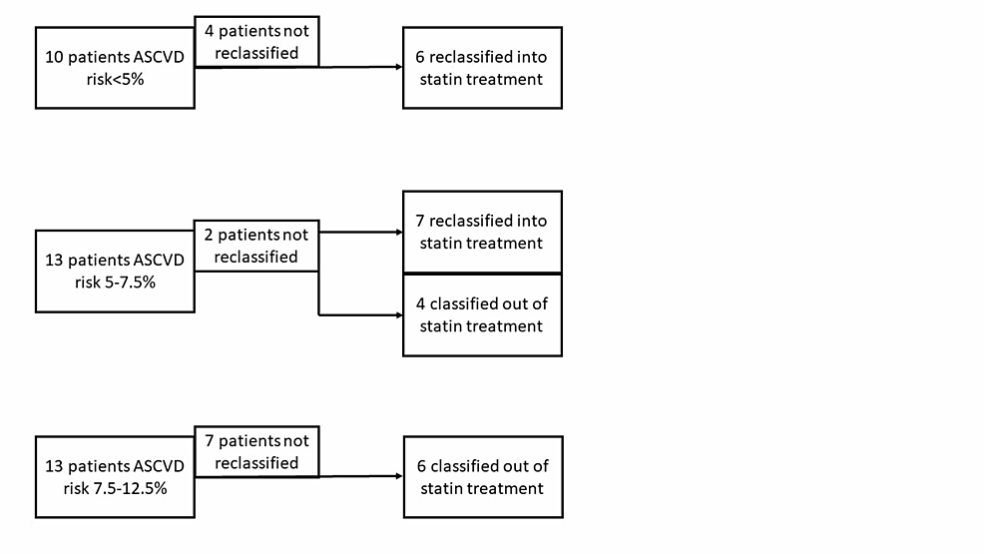
Reclassification process ASCVD, arteriosclerotic cardiovascular disease

A total of 521 scans were identified. After removing patients who were not between 40 and 75 years of age, 384 scans remained. After accounting for multiple scans on the same patient, 370 remained (Figure 1). Of these 370 patients who underwent ngCCT scans on one scanner within the study period, 36 patients met the inclusion criteria. In 63% of patients (23/36), CAC scoring altered statin management compared to ASCVD risk prediction alone. Thirteen patients had an ASCVD risk of 5%-7.5%. For the 13 intermediate-risk patients, 11 (85%) were able to be reclassified into higher or lower risk categories with the addition of CAC scoring. Of the 11 patients, seven were reclassified into the higher risk category and four were reclassified into the lower risk category. Thirteen patients had an ASCVD risk of >12.5%, of which six had a CAC score of 0. Finally, 10 patients had an ASCVD risk of <5%, of which six were recommended for statin treatment based on the Society of Cardiovascular Computed Tomography (SCCT) guidelines (Figure 2). Other scan information can be seen in Table 1 and Table 2.

**Table 1:** Scan demographics and phase of care ASCVD, arteriosclerotic cardiovascular disease; CAC, coronary artery calcium

	Inpatient	Outpatient	Total	Proportion confidence interval (95%)
Total scans by pooled cohort equation: 10-year ASCVD risk	19	17	36	NA
ASCVD risk < 5%	8	2	10	NA
Prescribe statin (CAC < 300 AU)	5	1	6/10 (60%)	[0.25, 0.95] 25-95%
ASCVD risk 5%-7%	4	9	13	NA
Deprescribe statin (CAC = 0 AU)	1	3	4/13 (31%)	[0.029, 0.587] 3-59%
Prescribe statin (CAC < 300 AU)	2	5	7/13 (54%)	[0.237, 0.839] 24-84%
ASCVD risk > 7.5%	7	6	13	NA
Deprescribe statin (CAC = 0 AU)	3	3	6/13 (46%)	[0.16, 0.762] 16-76%
Total scans by CAC			36	NA
CAC = 0 AU	7	7	14 (39%)	[0.231, 0.549] 23-55%
Indeterminant by CAC	3	1	4 (11%)	[0.008, 0.212] 1-21%
CAC > 300 AU	9	9	18 (50%)	[0.337, 0.663] 34-66%

**Table 2:** Scan demographics and scan methodology ASCVD, arteriosclerotic cardiovascular disease; CAC, coronary artery calcium; CAP, chest abdomen, and pelvis; CT, computed tomography

	CT chest without contrast	CT chest with and without contrast	Low-dose lung cancer screening	CT CAP without contrast	CT CAP with and without contrast	Total	Proportion confidence interval (95%)
Total scans by pooled cohort equation: 10-year ASCVD risk	17	1	8	8	2	36	NA
ASCVD risk < 5%	6	1	0	3	0	10	NA
Prescribe statin (CAC < 300 AU)	5	0	0	1	0	6/10 (60%)	[0.25, 0.95] 25-95%
ASCVD risk 5%-7.5%	4		6	2	1	13	NA
Deprescribe statin (CAC = 0 AU)	1	0	2	1	0	4/13 (31%)	[0.029, 0.587] 3-59%
Prescribe statin (CAC < 300 AU)	2	0	3	1	1	7/13 (54%)	[0.237, 0.839] 24-84%
ASCVD risk > 7.5%	7	0	2	3	1	13	NA
Deprescribe statin (CAC = 0 AU)	4	0	0	2	0	6/13 (46%)	[0.16, 0.762] 16-76%

## Discussion

This study evaluated CAC scores in patients without clinical ASCVD, DMII, or LDL > 190 mg/dL. In a similar population, numbers needed to treat to prevent a major adverse cardiovascular event within 10 years have ranged from thousands (if CAC score is 0), to 100 (CAC score is 1-99), to 12 (CAC is >100) [22]. CAC scans may assist in decreasing the number of statins prescribed, but if ordered to make that determination, it would not improve healthcare costs [13]. However, patients undergo ngCCTs for a variety of reasons, and the information about CAC in these scans can be used to help make treatment recommendations if it is reported with minimal additional cost. Using the information from these scans, better statin prescribing practices could be followed.

In this study, conservative guidelines were used for deprescribing statins with a CAC score of 0 AU. We were also conservative in overriding the pooled cohort equation in prescribing statins, setting our threshold at 300 AU. These thresholds were used in order to capture patients with a certain benefit or lack thereof, regardless of the ASCVD risk score. Even with these conservative thresholds, CAC scoring reclassified more than 60% of our patient population. The impact this has on health, cost, polypharmacy, and patient satisfaction can be substantial based on prior research [9,11-13,18,21].

Limitations of this study include a small sample size, retrospective nature, and single-center status. Since this is a retrospective study, many patients were excluded due to their ASCVD risk being incalculable. This primarily resulted from lack of a drawn lipid profile. However, if patients have access to primary care within the same system, this information can be obtained after the ngCCT was performed if the CAC score is still calculated. Ours was a single-center study, and for consistency across the measurements, only results from a single scanner were reviewed. Of the eligible patients, more than 50% had a clinical impact based on their CAC scores being reported. Finally, 95% confidence intervals were wide due to the small sample size of the study.

Future studies could show the benefit of this intervention across multiple sites. If integrated with primary care offices, the effects of reporting CAC scores on ngCCTs could have a profound impact on the prescription/deprescription of statins. This would be especially useful in the intermediate-risk ASCVD group (5%-7.5% 10-year risk). Also, as the pooled cohort equation evolves and is improved, CAC scores may provide a helpful additional piece of information for shared decision-making about statin use [21].

## Conclusions

This study describes a low-cost intervention, quantifying and classifying CAC scores on ngCCTs obtained for other reasons. This score can then be used to make decisions about statin utility in low- and intermediate-risk patients. Our study showed that a sizable proportion of the population may have statin recommendations changed if CAC scores are used to augment ASCVD risk in certain patients. The benefit of this intervention is well described in other literature but can be seen here to make a large clinical impact in a community hospital setting. Institution of this process can improve patient outcomes, satisfaction, and cost.
